# Twenty‐year survival outcomes after multipeptide vaccination for resected high‐risk melanoma: A post‐hoc analysis of a randomized clinical trial

**DOI:** 10.1002/ijc.70006

**Published:** 2025-06-19

**Authors:** Emily K. Ninmer, Hong Zhu, Kimberly A. Chianese‐Bullock, Craig L. Slingluff

**Affiliations:** ^1^ Department of Surgery/Division of Surgical Oncology and the Human Immune Therapy Center Cancer Center, University of Virginia Charlottesville Virginia USA; ^2^ Department of Public Health Sciences University of Virginia Charlottesville Virginia USA; ^3^ School of Medicine, Cancer Center University of Virginia Charlottesville Virginia USA

**Keywords:** immunotherapy, melanoma, peptide vaccine, shared antigens

## Abstract

In this post‐hoc analysis, we report long‐term clinical outcomes of a randomized phase II clinical trial (Mel39, NCT00938223) that tested the immunogenicity of two multipeptide vaccines designed to stimulate CD8^+^ T cells in patients with high‐risk melanoma. Fifty‐one participants with resected stage IIB‐IV melanoma randomized to vaccination with 4 or 12 melanoma peptides were followed for clinical outcomes. Overall survival (OS) and recurrence‐free survival (RFS) by vaccine arm, immune response, age, sex, and stage were evaluated. Median follow‐up was 16.1 years for all participants and 21.2 years for living participants. OS rates (95% CI) for both vaccine arms were 65% (51–78%) and 49% (35–63%) at 10 and 20 years, respectively, favoring vaccination with 12 melanoma peptides (HR 0.64, 95% CI: 0.29–1.40; *p* = .26) with a promising difference given the study sample size. Females had significantly improved RFS compared to males after either vaccine regimen, independent of peripheral immune response to the vaccine (HR 0.42, 95% CI: 0.19–0.91; *p* = .03). Overall, clinical efficacy was not significantly improved with more class I major histocompatibility complex (MHC)‐restricted peptides to the vaccine despite more favorable peripheral immune response rates on treatment. Females had durable RFS after vaccination that was not explained by sex‐associated differences in peripheral CD8^+^ T cell response rates during treatment. Further work to identify clinically meaningful vaccine‐induced T cell responses and how to optimize vaccines to elicit these responses is needed, including investigation into the influence of host factors on the response to immunotherapy.

## Graphical abstract

What's new?

While melanoma vaccines have shown therapeutic promise, predicting their clinical benefits is challenged by factors such as delayed response to immunotherapy and scarcity of biomarkers. Here, the authors examined the long‐term efficacy of vaccination with either 4 or 12 class I MHC‐restricted peptides in high‐risk melanoma. Long‐term survival following treatment with either vaccine regimen was favorable, with the addition of more MHC class I‐restricted peptides exhibiting no clear clinical benefit in melanoma patients. Durable RFS was observed among vaccine‐treated females, independent of peripheral CD8^+^ T cell responses, underscoring a need to better understand interactions between host factors and immunotherapy.

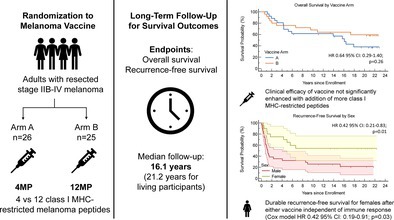

**Abbreviations**
4MP: four melanoma peptide vaccine
12MP: twelve melanoma peptide vaccine
AJCC: American Joint Committee on Cancer
CD4: cluster of differentiation 4
CD8: cluster of differentiation 8
CI: confidence interval
ECOG PS: Eastern Cooperative Oncology Group Performance Status
GM‐CSF: granulocyte‐macrophage colony‐ stimulating factor
HLA: human leukocyte antigen
HR: hazard ratio
IFN: interferon
IVS: in‐vitro stimulated
MHC: major histocompatibility complex
OS: overall survival
PBMC: peripheral blood mononuclear cell
RFS: recurrence‐free survival
SIN: sentinel immunized node

## 1 INTRODUCTION

Melanoma vaccines have had promising results in early phase clinical trials.^1^, ^2^, ^3^, ^4^ Improving clinical efficacy requires optimizing antigen selection, adjuvants, and delivery platforms, yet there are few randomized trials designed to assess the impact of these vaccine components on outcomes. Also, identifying patients who may receive the greatest benefit from cancer vaccines remains elusive. Potential biomarkers to predict response to cancer vaccines have not been well characterized, and the experience with immune checkpoint blockade suggests that multiple biomarkers are needed to characterize the dynamics of the antitumor response.^5^ The challenge in predicting clinical benefit with cancer vaccines also reflects limitations of survival analysis of immunotherapeutic agents that may produce delayed, yet durable responses.^6^

One strategy to optimize cancer vaccines is to incorporate more antigens to overcome loss of antigen expression and major histocompatibility complex (MHC) expression, two mechanisms of tumor immune escape.^7^, ^8^, ^9^ We previously designed a single center, phase II randomized trial to compare the immunogenicity of vaccination with 12 melanoma peptides (12MP) vs. 4 melanoma peptides (4MP) (Mel39, NCT00938223).^10^ The vaccines were comprised of class I MHC‐restricted peptides derived from melanocyte differentiation proteins and cancer‐testis antigens. We hypothesized that vaccination with 12MP would produce a broader and more robust immune response compared to 4MP, without significant inhibition from peptide competition for MHC binding. Both hypotheses were supported by the original immune analysis, but assessment of clinical outcomes was limited by short follow‐up.^10^

The objective of the present study was to assess long‐term clinical outcomes of participants in this trial. We hypothesized that participants vaccinated with 12MP would have improved survival outcomes compared to those vaccinated with 4MP and that participants who had an immune response, regardless of vaccine regimen, would have improved survival outcomes. We also performed exploratory analyses to identify subgroups with the greatest clinical benefit after multipeptide vaccination.

## 2 METHODS

### 2.1 Study design

This post‐hoc analysis included all eligible participants enrolled on the Mel39 trial, for which the study design and detailed methods have been reported.^10^ Adult patients with resected stage IIB‐IV melanoma (American Joint Committee on Cancer; AJCC; 6th edition^11^) arising from cutaneous, mucosal, or unknown sites were eligible for inclusion. Eligibility required expression of HLA‐A1, ‐A2, or ‐A3 by serological assay, as restricting MHC for the vaccine peptides, and the expression of gp100 and/or tyrosinase by the tumor. Patients were randomized 1:1 to receive 4MP (Arm A) or 12MP (Arm B), stratified by AJCC stage. A class II MHC‐restricted tetanus toxoid peptide was included in both formulations for CD4^+^ T cell activation.^12^ Vaccine peptide mixtures were administered in an emulsion of granulocyte‐macrophage colony stimulating factor (GM‐CSF) and Montanide ISA‐51 adjuvant.

**FIGURE 1:**
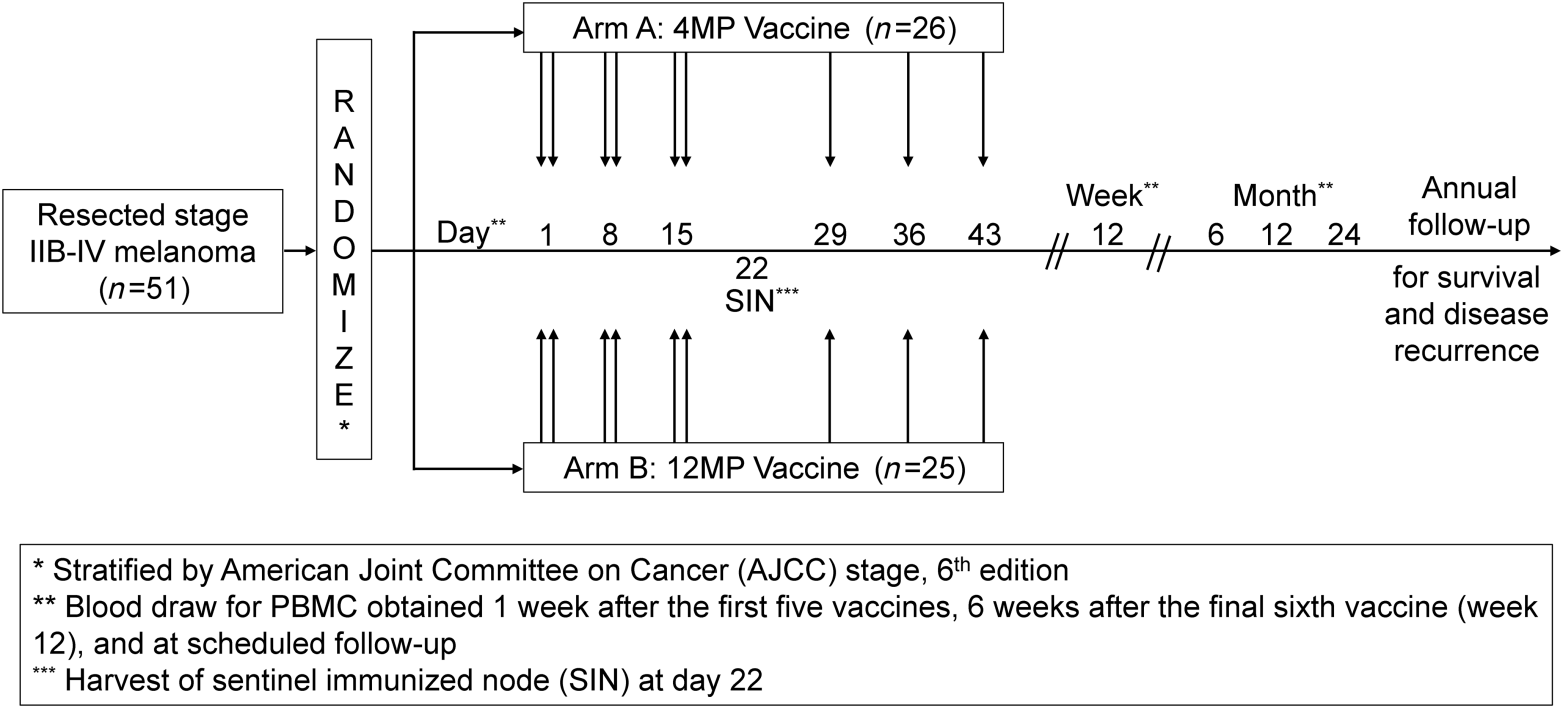
Trial schema. Patients with resected stage IIB‐IV melanoma with expression of HLA‐A1, ‐A2, ‐A3 were randomized to receive one of two multipeptide vaccines: 4MP or 12MP. Both formulations contained a tetanus toxoid peptide with granulocyte‐macrophage colony stimulating factor (GM‐CSF) and Montanide ISA‐51 as adjuvant. Vaccines were administered at day 1, 8, 15, 29, 36, and 43. A replicate vaccine was given at a different site on days 1, 8, and 15 followed by harvest of a lymph node draining this replicate site (sentinel immunized node; SIN) at day 22. Circulating response to vaccine was measured in peripheral blood mononuclear cells (PBMC).

The trial schema is shown in Figure 1. Vaccines were administered subcutaneously and intradermally on days 1, 8, 15, 29, 36, and 43. Blood samples for immune analysis (peripheral blood mononuclear cells, PBMC) were collected 1 week after each of the first five vaccines and 6 weeks after the final (sixth) vaccine. Participants received a replicate vaccine in a different extremity on days 1, 8, and 15, followed by harvest of a lymph node draining this site (sentinel immunized node, SIN) on day 22. PBMC and SIN samples were evaluated for CD8^+^ T cell responses at the time of the original analysis using in‐vitro stimulated (IVS) interferon‐gamma (IFN‐γ) ELIspot assay as previously described.^13^ Participants were followed for clinical exam, laboratory, and imaging studies at 3, 6, 12, and 24 months, and annually thereafter for clinical outcomes. All participants were enrolled and treated from 2001 to 2003. Data analyses for the present study were performed from June 2023 to March 2025.

### 2.2 Data and outcomes

Data were retrieved from the University of Virginia OnCore database, Cancer Center Clinical Trials Office database, and associated clinical records. Demographic data included biologic sex and age at enrollment. In a previous analysis of three different shared antigen peptide vaccine trials by our group, we found that immune response rates were significantly lower in patients aged 64+ years compared to patients less than 64 years old.^14^ For the present study, participants were grouped by age as either older (65+ years) or younger (<65 years). Clinical data included Eastern Cooperative Oncology Group performance status (ECOG PS), AJCC stage, and disease status (initial vs. recurrent), as patients were rendered clinically free of disease from either their initial diagnosis or recurrence prior to enrollment. For this analysis, disease stage was re‐classified according to the AJCC 8th edition criteria.^15^ Immunologic data included HLA type and the presence of multiple restricted HLA alleles, as well as immune response (yes vs. no) in the PBMC and SIN. For the present study, analyses of the PBMC were limited to samples through 6 months due to inconsistent sample collection beyond 6 months. A persistent immune response to month 6 was defined as an immune response detected during the vaccine series (by day 50) and also at 6 months.

All participants were followed for survival and disease recurrence. Endpoints for this analysis were overall survival (OS) and recurrence‐free survival (RFS). OS was measured from study entry to last known follow‐up or date of death. For living participants, the last known follow‐up for OS was defined as the most recent date of either the last study annual follow‐up or any interaction with the healthcare system. RFS was measured from study entry to date of first recurrence, including new primary melanomas, or date of last known disease status; participants who died without recurrence were censored for RFS at the date of last known disease status.^16^ Given the long follow‐up, we did not include death from any cause as an event for RFS, as deaths from causes unrelated to melanoma were expected and captured for OS. For participants without recurrence, the date of last known disease status was defined as the most recent date of either the last study annual follow‐up or last follow‐up with an oncologist, or dermatologist, or last diagnostic imaging ordered by any healthcare provider to evaluate for recurrent disease.

### 2.3 Statistical analyses

Comparisons of participant characteristics between groups were performed using the Mann–Whitney test for continuous variables and Chi‐square test or Fisher's exact test as appropriate for categorical variables. Using intention‐to‐treat analysis, OS and RFS estimates were obtained by the Kaplan–Meier method with between‐group comparisons using the log‐rank test. Outcomes by immune response were evaluated at landmark points: day 50 and month 6 for PBMC samples after completion of the vaccination series and at 6‐month follow‐up, and day 22 for SIN lymphocytes after the lymph node harvest. Cox regression analyses were performed to investigate the association of clinical and immunologic factors with the survival endpoints. A two‐sided *p*‐value <.05 was considered statistically significant. A power calculation was performed using PASS 2023 to demonstrate the detectable hazard ratios for binary covariates with the available study sample size to provide at least 80% power at a two‐sided .05 significance level for the survival endpoints (OS and RFS), based on the Cox regression model. Fisher's exact tests for comparisons larger than 2 × 2 were calculated using Stata version 18 (StataCorp, College Station, TX). All other statistical analyses were performed using MedCalc Statistical Software version 22.030 (MedCalc Software Ltd., Ostend, Belgium).

## 3 RESULTS

### 3.1 Baseline characteristics and clinical course

Data were collected for all 51 eligible participants, including 26 on Arm A (4MP) and 25 on Arm B (12MP). The median follow‐up interval was 16.1 years for all participants and 21.2 years for participants alive at last known follow‐up. Fourteen (27%) were age 65+ years. The trial enrolled more males (61%) than females (39%) in proportions representative of the melanoma patient population.^17^ Median ages were 56.2 and 48.0 years for males and females, respectively, also representative of the melanoma patient population.^17^

Demographic and clinical characteristics by vaccine arm were well matched (Table 1), but there were trends on Arm A to younger age (median age 48.7 vs. 56.2 years; *p* = .78) and fewer males (54% vs. 68%; *p* = .31). HLA‐A1 and HLA‐A2 expression was similar on both arms, while there was greater HLA‐A3 expression on Arm B (38% vs. 64%; *p* = .07). Of participants who expressed HLA‐A3, 14 (54%) also expressed HLA‐A1 or HLA‐A2. On each arm, approximately 70% had stage III disease by AJCC 6th edition criteria used for stratified randomization at enrollment (Table S1). Stage remained balanced across arms with re‐staging by AJCC 8th edition criteria. Recurrent disease prior to enrollment trended to be more common on Arm B (35% vs. 52%; *p* = .21). Participant characteristics were generally balanced by age group and sex (Tables S2 and S3), though 7 of the 8 participants with stage IV disease were younger males.

**TABLE 1:** Patient characteristics by vaccine arm.

	Arm A (*n* = 26)	Arm B (*n* = 25)	Total (*n* = 51)	*p* value^a^
Median age, years (range)	48.7 (28.2–75.0)	56.2 (30.3–82.1)	53.1 (28.2–82.1)	.78
Male, *n* (%)	14 (54%)	17 (68%)	31 (61%)	.31
Class I MHC allele^b^, *n* (%)				
HLA‐A1	10 (38%)	6 (24%)	16 (31%)	.27
HLA‐A2	15 (58%)	13 (52%)	28 (55%)	.69
HLA‐A3^c^	10 (38%)	16 (64%)	26 (51%)	.07
Multiple HLA alleles^d^, *n* (%)	9 (35%)	10 (40%)	19 (37%)	.69
Disease status, *n* (%)				
Initial diagnosis	17 (65%)	12 (48%)	29 (57%)	.21
Recurrent diagnosis	9 (35%)	13 (52%)	22 (43%)	
AJCC stage, *n* (%)				
II	4 (15%)	4 (16%)	8 (16%)	.77
III	19 (73%)	16 (64%)	35 (69%)	
IV	3 (12%)	5 (20%)	8 (16%)	
ECOG PS score 0, *n* (%)	19 (73%)	21 (84%)	40 (78%)	.50

Abbreviations: AJCC, American Joint Committee on Cancer (8th edition); ECOG PS, Eastern Cooperative Oncology Group Performance Status; HLA, human leukocyte antigen; MHC, major histocompatibility complex.

^a^ Chi‐square test and Fisher's exact test (counts of *n* ≤ 5) for categorical variables; Mann–Whitney test for age; significant *p* < .05.

^b^ Counts represent the number of restricted alleles. Patients may express two different restricted alleles, resulting in counts greater than the sample size (>100%).

^c^ HLA‐A3 superfamily, including HLA‐A3, HLA‐A11, HLA‐A31.

^d^ Counts represent the number of participants who express two different restricted alleles.

Thirty‐three (65%) participants had at least one recurrence after enrollment, with 17 (52%) on Arm A and 16 (48%) on Arm B. For these participants who developed recurrence, median (range) time to first recurrence after enrollment was 1.03 (0.02–15.27) years, and 23 (70%) died, with median (range) time from first recurrence after enrollment to death of 1.46 (0.12–16.56) years. Median (range) time from first recurrence after enrollment to last known follow‐up for living participants was 20.36 (6.64–22.61) years. Characteristics of participants with recurrence after enrollment are summarized by vaccine arm in Table S4.

### 3.2 Immune response

Immune response rates in the SIN and PBMC are summarized by vaccine arm and by two host factors, sex and age, in Table 2. Forty‐seven (92%) participants had evaluable SIN samples, among whom 19 (73%) on Arm A and 22 (100%) on Arm B had an immune response. Only 6 participants in total did not have an immune response in the SIN. Immune response rates in the SIN were higher for females and younger participants.

**TABLE 2:** Immune response rates by vaccine arm, sex, and age group.

	SIN Any IR (*n* = 47)	PBMC IR by Day 50 (*n* = 50)	PBMC Persistent IR to Month 6^a^ (*n* = 31)
By Vaccine Arm			
A	19/25 (73%)	18/26 (69%)	8/20 (40%)
B	22/22 (100%)	20/24 (83%)	4/11 (36%)
By Sex			
Female	18/19 (95%)	15/19 (79%)	5/13 (38%)
Male	23/28 (82%)	23/31 (74%)	7/18 (39%)
By Age Group			
<65 Years	31/33 (94%)	31/36 (86%)	9/20 (45%)
65+ Years	10/14 (71%)	7/14 (50%)	3/11 (27%)

Abbreviations: IR, immune response; PBMC, peripheral blood mononuclear cells; SIN, sentinel immunized node.

^a^ Persistence is defined as an immune response during the vaccine treatment (by day 50) in addition to an immune response at month 6 follow‐up.

Fifty participants (98%) had evaluable PBMC samples by day 50 (after the first five vaccines), among whom 18 (69%) on Arm A and 20 (83%) on Arm B had an immune response (Table 2). By day 50, the median number of time points per participant with an immune response that met positivity criteria was 4; only 5 (13%) participants had a response at only a single time point. Immune response rates were inversely associated with age, but did not differ by sex. Forty‐seven (92%) participants had evaluable PBMC samples at week 12 (6 weeks after the final sixth vaccine), of whom 21 (45%) had an immune response (data not shown). All participants with an immune response at week 12 had previously met criteria at least once for an immune response by day 50. Of the 38 participants in total who had an immune response in PBMC, two (5%) did not have an immune response in the SIN, and two (5%) did not have evaluable SIN samples. One participant had recurrence before day 50, and thus was not evaluable on landmark analysis for RFS by immune response by day 50.

Thirty‐one (61%) participants had evaluable PBMC at the 6‐month follow‐up (Table 2). While higher immune response rates were observed on Arm B during treatment, similar rates were observed for either arm in those with evaluable PBMC at month 6. Immune response rates during treatment and at the 6‐month follow‐up were similar for both sexes. Younger participants had higher immune response rates during treatment compared to older participants, with a similar trend of higher persistent immune response rates among those with evaluable PBMC at month 6. Eight participants had recurrence prior to month 6, and thus were not evaluable on landmark analysis for RFS by persistent immune response to month 6.

### 3.3 Overall survival

**FIGURE 2:**
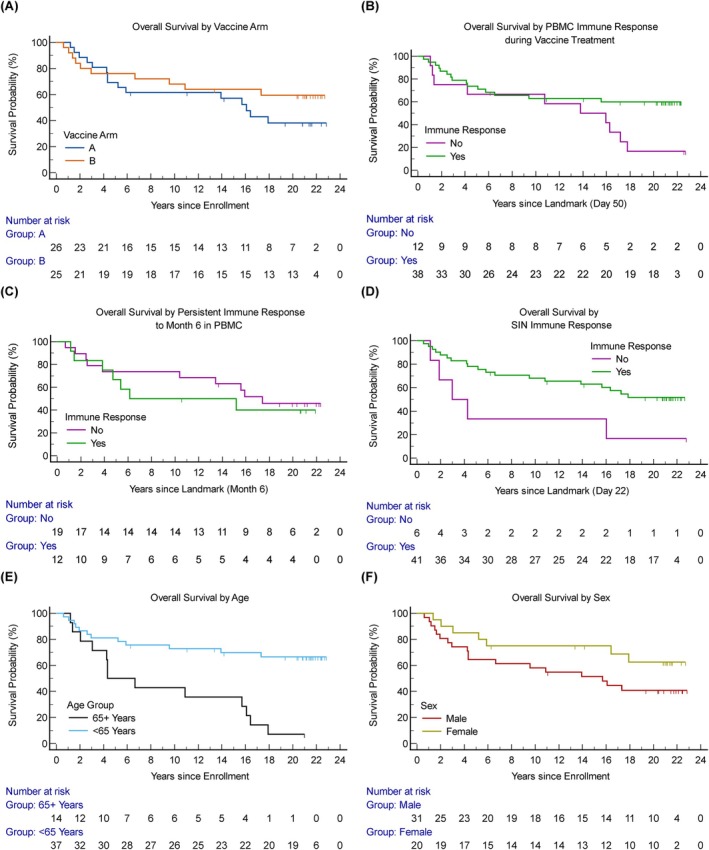
Overall survival. Kaplan–Meier overall survival (OS) curves by (A) vaccine arm (HR 0.64 95% CI: 0.29–1.40; *p* = .26), (B) immune response in peripheral blood mononuclear cells (PBMC) during vaccine treatment using landmark at day 50 (HR 0.36 95% CI: 0.14–0.92; *p* = .03), (C) persistent immune response in PBMC using landmark at month 6 (HR 1.34 95% CI: 0.49–3.65, *p* = .57), (D) immune response in the sentinel immunized node (SIN) using landmark at day 22 (HR 0.22 95% CI: 0.05–0.90; *p* = .03), (E) age group (HR 0.16 95% CI: 0.06–0.42; *p* = .0002), and (F) sex (HR 0.53 95% CI: 0.24–1.17; *p* = .12).

The OS rates (95% CI) for both vaccine arms combined were 65% (51–78%) and 49% (35–63%) at 10 and 20 years, respectively (Figure S1A). The OS rate estimates at 5‐year intervals by vaccine arm and for both vaccine arms combined are provided in Table S5. Median OS (95% CI) for the 4MP and 12MP vaccine arms was 16.1 (4.3–17.9) years and not reached, respectively. A weak trend to improved OS after vaccination with 12MP compared to 4MP was observed (HR 0.64, 95% CI: 0.29–1.40; *p* = .26; Figure 2A). Our power calculation suggests that the study sample size would provide at least 80% power at a two‐sided .05 significance level to detect a HR of 0.32 for a binary covariate (e.g., vaccine arm), given the event rate of 49%, and it only provides about 20% power to detect a significant difference in OS with the estimated HR of 0.64 for the vaccine arm. The sample size needed for 80% power to detect a significant difference in OS by vaccine arm (HR of 0.64) at a two‐sided .05 significance level was estimated to be 323 patients (about 162 per arm).

Participants with a PBMC immune response to either vaccine during treatment had similar OS rates to year 14, with progressive widening of the curves thereafter, resulting in significantly improved OS for those with an immune response compared to those without (median OS (95% CI): not reached vs. 13.8 (1.2–17.8) years; HR 0.36, 95% CI: 0.14–0.92; *p* = .03; Figure 2B). However, no significant difference in OS by persistent immune response to month 6 was found among participants who had evaluable PBMC at month 6 (HR 1.34 95% CI: 0.49–3.65, *p* = .57; Figure 2C). Participants with a SIN immune response to either vaccine had significantly improved OS compared to those without a response (median OS (95% CI): not reached vs. 3.0 (1.1–16.0) years; HR 0.22, 95% CI: 0.05–0.90; *p* = .03; Figure 2D).

Exploratory analyses were performed to evaluate OS by age, sex, and AJCC stage after either vaccine regimen. Younger participants had significantly improved OS compared to older participants (median OS (95% CI): not reached vs. 4.3 (2.0–16.1) years; HR 0.16, 95% CI: 0.06–0.42; *p* = .0002; Figure 2E) and there was a trend to improved OS for females compared to males (median OS (95% CI): not reached vs. 15.7 (4.3–17.3) years; HR 0.53, 95% CI: 0.24–1.17; *p* = .12; Figure 2F). There were no significant differences in OS by AJCC stage (Figure S2A).

Given the imbalance in immune response rates in PBMC by age group, the association of age and PBMC immune response during treatment with OS was evaluated by Cox regression analysis using a landmark at the end of treatment (day 50). There were 25 events in this analysis, so only two covariates were assessed in the Cox regression analysis. PBMC immune response to either vaccine during treatment was not a significant predictor of OS after adjusting for age (PBMC immune response HR 0.73, 95% CI: 0.30–1.77; *p* = .48; Table 3).

**TABLE 3:** Cox regression models for landmark analysis of clinical outcomes at end of treatment (day 50).

Covariate	Detail	HR	95% CI	*p* value^a^
**Model of overall survival with PBMC immune response and age (Chi‐squared 11.417, *p* = .0033) (*n* = 50)**				
Age	65+ (ref) vs. <65 years	0.29	0.12–0.69	.**0056**
PBMC immune response	No (ref) vs. Yes	0.73	0.30–1.77	.4819
**Model of recurrence‐free survival with PBMC immune response and sex (Chi‐squared 7.465, *p* = .0239) (*n* = 49)**				
Sex	Male (ref) vs. female	0.42	0.19–0.91	.**0287**
PBMC immune response	No (ref) vs. Yes	0.54	0.26–1.15	.1110

Abbreviations: CI, confidence interval; HR, hazard ratio; PBMC, peripheral blood mononuclear cells; ref, reference value.

^a^ Significant *p* < .05 (bolded).

### 3.4 Recurrence‐Free Survival

**FIGURE 3:**
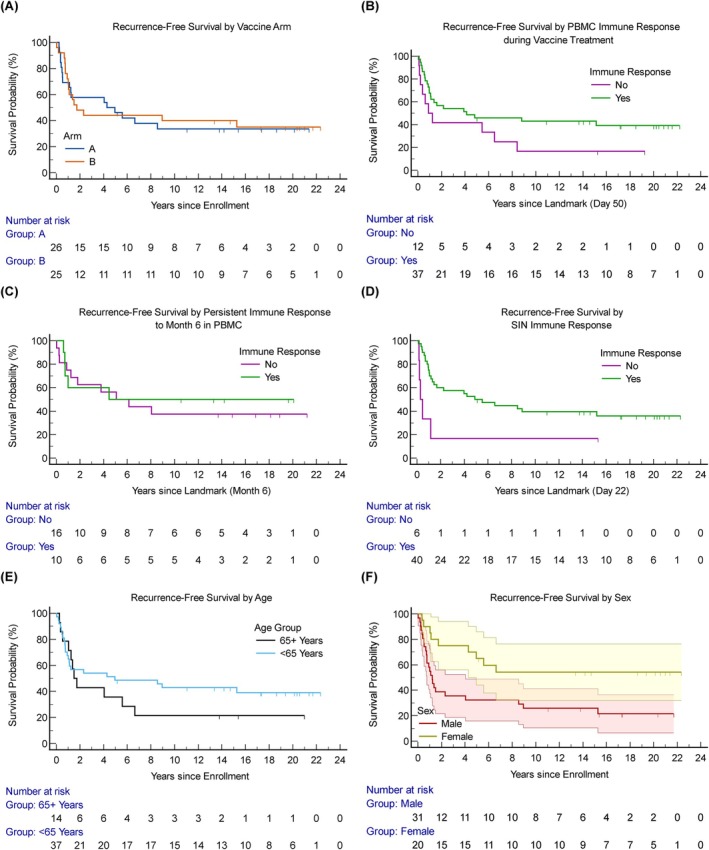
Recurrence‐free survival. Kaplan–Meier recurrence‐free survival (RFS) curves by (A) vaccine arm (HR 0.93 95% CI: 0.47–1.85; *p* = .85), (B) immune response in peripheral blood mononuclear cells (PBMC) during vaccine treatment using landmark at day 50 (HR 0.50 95% CI: 0.21–1.19; *p* = .12), (C) persistent immune response in PBMC using landmark at month 6 (HR 0.81 95% CI: 0.28–2.28, *p* = .68), (D) immune response in the sentinel immunized node (SIN) using landmark at day 22 (HR 0.18 95% CI: 0.04–0.77; *p* = .02), (E) age group (HR 0.68 95% CI: 0.31–1.49; *p* = .34), and (F) sex (HR 0.42 95% CI: 0.21–0.83; *p* = .01).

Most recurrences in the study population occurred early, with few recurrences after 8 years (Figure S1B). Median RFS (95% CI) for the 4MP and 12MP vaccine arms was 4.3 (0.5–8.6) years and 1.7 (1.0–15.3) years, respectively. There was no significant difference in RFS by vaccine arm (HR 0.93 95% CI: 0.47–1.85; *p* = .85; Figure 3A). For participants without recurrence by 2 years, the estimated conditional probability rates of remaining disease‐free in 5 years (i.e., at 7 years after enrollment) were 66% and 92% for Arms A and B, respectively, and the estimated conditional probability rates of remaining disease‐free in 10 years (i.e., at 12 years after enrollment) were 58% and 83% for Arms A and B, respectively.

A favorable trend to improved RFS was found for participants with a PBMC immune response to either vaccine (median RFS (95% CI): 4.1 (1.1–15.1) vs. 0.9 (0.1–8.4) years; HR 0.50, 95% CI: 0.21–1.19; *p* = .12; Figure 3B). However, no significant difference in RFS by persistent immune response to month 6 was found among participants who had evaluable PBMC at month 6 (HR 0.81 95% CI: 0.28–2.28, *p* = .68; Figure 3C). Participants with a SIN immune response to either vaccine had significantly improved RFS compared to those without a response (median RFS (95% CI): 0.3 (0.2–1.2) vs. 4.9 (1.3–15.2) years; HR 0.18, 95% CI: 0.04–0.77; *p* = .02; Figure 3D).

Exploratory analyses were performed to evaluate RFS by age, sex, and AJCC stage after either vaccine regimen. There was a weak trend to improved RFS for younger participants (median RFS (95% CI): 5.0 (1.0–15.3) vs. 1.5 (0.5–6.6) years; HR 0.68, 95% CI: 0.31–1.49; *p* = .34; Figure 3E). Females had significantly improved RFS compared to males (median RFS (95% CI): not reached vs. 1.2 (0.7–4.0) years; HR 0.42, 95% CI: 0.21–0.83; *p* = .01; Figure 3F). There were no recurrences in females after 7 years, with stable RFS at 54% (95% CI: 32–77%) to beyond 20 years and a 33‐percentage point difference in RFS between males and females after 15 years. There were no significant differences in RFS by AJCC stage (Figure S2B).

The association of PBMC immune response during treatment and sex with RFS was evaluated by Cox regression analysis using a landmark at the end of treatment (day 50). For this analysis, there were 32 events, so we limited the Cox regression analysis to just two covariates. Female sex remained a significant predictor of RFS after adjusting for the PBMC immune response rate during treatment (female sex HR 0.42, 95% CI: 0.19–0.91; *p* = .03; Table 3). From our power calculation to determine the detectable HR for RFS with our sample size and event rate based on the Cox regression model, an HR of 0.37 for a binary covariate, such as sex, provides at least 80% power at a two‐sided .05 significance level.

## 4 DISCUSSION

We report a post‐hoc analysis of the Mel39 trial, a single‐center, phase II randomized trial that enrolled participants with resected high‐risk melanoma to receive one of two multipeptide melanoma vaccines. The trial was powered to evaluate for differences in immunogenicity of the two vaccine regimens. However, these data provided an opportunity to explore the impact of adding more class I MHC‐restricted antigens to vaccines on long‐term clinical outcomes and to identify subgroups that had the greatest clinical benefit after either vaccine regimen. We found a promising trend to improved OS for participants vaccinated with 12MP compared to 4MP, but this analysis was underpowered to detect a significant difference at the estimated HR of 0.64. Thus, these findings are consistent with a better outcome with 12MP, but would need to be definitively tested in a larger randomized trial powered to detect differences in survival. There was a significant association between PBMC immune response and OS on univariable analysis, but this did not remain after adjustment for age. The significant associations between the SIN immune response and clinical outcomes on univariable analyses are promising, but the small number of participants without an immune response in the SIN limited multivariable analyses.

Participants on both arms had favorable long‐term survival. This trial reflects the treatment paradigms in place prior to the use of checkpoint blockade therapies. At the time that participants were enrolled in this trial, systemic interferon (IFN) was an approved adjuvant therapy for patients with resected high‐risk melanoma. The OS rates in this trial compare favorably to those reported in randomized controlled trials for systemic IFN therapy. In the ECOG 1684 trial that randomized patients with resected stage IIB–III (AJCC 6th edition) melanoma to treatment with IFN alfa‐2b vs. observation, the 5‐year OS rates (95% CI) were 46% (39–55%) and 37% (30–46%), respectively.^18^ In the EORTC 18991 trial that randomized patients with resected stage III (AJCC 6th edition) melanoma to treatment with IFN alfa‐2b vs. observation, the 7‐year OS rates (standard error) were 47.8% (2.0%) and 46.4% (2.0%), respectively.^19^ In our study, 5‐ and 7‐year OS rates (95% CI) after vaccination with either regimen were 73% (60–85%) and 67% (53–80%), respectively. These 5‐ and 7‐year OS rates exceed those of the observation and treatment groups in the ECOG and EORTC trials without overlap of 95% CIs. The OS rates by vaccine arm (Table S5) also compare favorably to those of both the treatment and observation groups in the ECOG and EORTC trials. Additionally, a pooled analysis of the ECOG 1684 trial and a similar ECOG 1690 trial^20^ demonstrated long‐term OS rates for both the IFN‐treated and observation groups of around 40% at both 15‐year and 20‐year intervals,^21^ which is less than the long‐term OS rates observed in this study for either vaccine arm at 15 years and arm B (12MP) at 20 years. Comparison of outcomes between different clinical trials should be interpreted with caution; however, the long‐term outcomes from our study raise the possibility that each vaccine regimen may have had a favorable clinical benefit.

There were few late recurrences for participants on this trial. For females, there were no recurrences after 7 years, with a plateau of the RFS curve at 54% to beyond 20 years. In a large cohort of patients with melanoma treated with curative intent and who remained disease‐free for at least 10 years, actuarial risks of late recurrence at 15 and 20 years were 6.8% and 11.3%, respectively.^22^ In that cohort, there were no significant differences in late recurrences by sex on multivariable analysis, though females were more likely to have late recurrence on univariable analysis.^22^ Thus, the long plateau of the RFS curve for females is unexpected, suggesting that multipeptide vaccines may contribute to long‐term RFS, which could enhance cure rates for some patients with resected melanoma.

We found that females had significantly improved RFS after multipeptide vaccination, and our power calculation suggests that the study sample size provides generally adequate power to detect the HR for this binary variable estimated by the Cox regression model. This difference in RFS by sex was not explained by differences in peripheral CD8^+^ T cell response to the vaccine during treatment. However, this analysis may not reflect all relevant differences in immune responses: females have a greater number of circulating CD4^+^ T cells and generate stronger antibody responses to vaccines than males.^23^ In a different study, we previously found that vaccination with 12MP plus six melanoma‐specific helper peptides (6MHP) designed to stimulate both CD8^+^ and CD4^+^ T cells was associated with improved long‐term survival in males, but not in females, when compared to vaccination with 12MP plus the tetanus toxoid peptide.^4^ In that study, there were also no sex differences in peripheral T cell response rates to vaccine,^24^ and antibody responses were not assessed. In the context of those findings, the results from the present study suggest that future optimization of melanoma vaccines to elicit clinically meaningful T cell responses to the vaccine may be best focused on enhancing CD4^+^ T cell responses to melanoma antigens. Additionally, prior work in vaccines to prevent infectious disease has revealed sex‐based differences in clinical efficacy not explained by vaccine‐induced immune responses.^25^ Our findings highlight the need for further investigation to better characterize sex differences in the measures of immunogenicity and clinical efficacy of cancer vaccines.

Alternatively, it is possible that the favorable RFS for females in this trial is independent of the benefit from the vaccine. Female sex has been identified as a favorable prognostic factor across all stages of melanoma.^26^, ^27^, ^28^, ^29^, ^30^ The significantly improved RFS for females after multipeptide vaccination in our trial is consistent with sex‐based survival differences found in meta‐analyses of larger randomized controlled EORTC trials in melanoma patient populations similar to that of our trial. Specifically, a 30% relative survival advantage for females in early‐stage disease^31^ and a 15–20% relative survival advantage in advanced disease^32^ have been observed, with persistence across age groups, across multiple clinical endpoints after adjusting for other prognostic factors and treatment. The extent to which this female survival advantage reflects biological sex differences in the spontaneous antitumor response, the response to immunotherapy, or both has not been well established. The impact of differential sex‐linked gene expression and sex steroid signaling on the innate and adaptive immune system has been proposed to underlie sex differences in the antitumor response^33^, ^34^, ^35^ and the response to immunotherapies.^36^, ^37^ Analyses of the melanoma tumor microenvironment have revealed sex differences in the immune cell infiltrate,^38^ mutation burden,^39^, ^40^ and immune evasion mechanisms,^41^ all of which may contribute to sex‐based differences in clinical outcomes and response to immunotherapies. However, sex‐specific differences in endpoints in oncology trials are inconsistently reported, with these comparisons often evaluated by meta‐analyses across multiple cancers and treatment regimens.^42^ Thus, future immuno‐oncology trials in melanoma should report safety, immunologic, and clinical endpoints by sex and, for the design of phase III trials, consider stratification by sex.

Our data reveal trends in the association of age and immune response to vaccine consistent with prior analyses comparing immune response rates to similar shared antigen vaccines by age.^14^ Older participants (65+ years) had lower CD8^+^ T cell response rates in the PBMC, particularly during vaccine treatment, and in the SIN. Our data are consistent with prior reports that identify age as an important prognostic factor across all stages of melanoma.^26^, ^27^ Decline in immunologic function with age impacts innate and adaptive immunity, including impaired dendritic cell function^43^, ^44^ and diversity of T cell responses.^45^, ^46^ With vaccines to prevent infections, lower efficacy has been reported in the elderly.^47^, ^48^, ^49^ Differences in the immunogenicity and efficacy of cancer vaccines due to age, particularly as it relates to modulating the influence of other host factors on immune function, have not been well characterized and warrant further investigation.

This study has several limitations. The original study was not powered to test differences in clinical outcome. Also, due to the small sample size, slight imbalances in participant characteristics by vaccine arm limit the interpretation of the results for outcomes by treatment groups, in addition to the lack of a control group. The small sample size prevented the analysis of all relevant prognostic factors in a multivariable model. While we were able to construct models to explore the association of peripheral immune response during treatment and either age or sex on outcome, these analyses are limited. While most participants had PBMC CD8^+^ T cell responses at multiple weeks on treatment, analysis of the other immunologic endpoints was limited. Deeper immune analyses of the SIN were not performed. Also, CD4^+^ T cell responses were not measured, and re‐challenge for a memory T cell response during follow‐up was not performed. Analyses of immune responses may be enhanced by evaluating for more than just IFN‐γ secretion, and by also evaluating vaccine‐induced T cells for multifunctionality and for ability to infiltrate metastases. Given the limitations in sample size and measured immune features, our results may reflect differences in overall immunologic fitness rather than vaccine‐induced immunity, though the influence of sex and age on response to immunotherapy remains an important area of further investigation. Data on BRAF mutation, PD‐L1 expression, and tumor mutation burden were not included, as enrollment preceded routine testing for these features. Finally, we used a pragmatic approach to assess RFS by participant report or documentation of an assessment (clinical exam or imaging) in the medical record, as scheduled imaging is not routine beyond 5 years.

In summary, this post‐hoc analysis of patients with resected high‐risk melanoma revealed favorable long‐term survival after treatment with either vaccine regimen, though the addition of more class I MHC‐restricted peptides to the vaccine did not significantly enhance clinical efficacy. We found evidence of durable RFS for females after multipeptide vaccination that was not explained by differences in peripheral CD8^+^ T cell response rates during treatment. However, a comprehensive assessment of vaccine immunogenicity with correlative studies to characterize other features of vaccine‐induced immune activity may be more informative. Analyses for the association of immune response with clinical outcome also require sufficient enrollment to support multivariable analyses to adjust for host factors that may be associated independently with immune response and outcome. Thus, further work to identify clinically meaningful vaccine‐induced T cell responses and how to optimize vaccines to elicit these responses is needed, including investigation into the influence of host factors on the response to immunotherapy.

## AUTHOR CONTRIBUTIONS

**Emily K. Ninmer:** Conceptualization; formal analysis; writing – original draft; writing – review and editing. **Hong Zhu:** Formal analysis; writing – review and editing. **Kimberly A. Chianese‐Bullock:** Data curation; writing – review and editing. **Craig L. Slingluff Jr.:** Conceptualization; resources; data curation; formal analysis; supervision; funding acquisition; methodology; writing – review and editing.

## FUNDING INFORMATION

The trial was supported by NCI R01 CA57653 (C.L. Slingluff, Jr.); the Cancer Research Institute by provision of infrastructure support for the University of Virginia Human Immune Therapy Center; the University of Virginia Cancer Center Support Grant (NCI P30 CA44579, Clinical Trials Office, Tissue Procurement Facility, Biomolecular Core Facility); the University of Virginia General Clinical Research Center (NIH grant 5 M01 RR00847); the Pratt Fund at the University of Virginia; and a generous philanthropic gift from Mr. and Mrs. William Goodwin; the Rebecca Harris Fellowship (E.K.N.). C.L.S. has served as sponsor‐investigator, holding the IND on behalf of the University of Virginia. There was no other external sponsor.

## CONFLICT OF INTEREST STATEMENT

C.L.S. has the following disclosures: Research support to the University of Virginia from Celldex (funding, drug), Glaxo‐Smith Kline (funding), Merck (funding, drug), 3 M (drug), Theraclion (device staff support); Funding to the University of Virginia from Polynoma for PI role on the MAVIS Clinical Trial; Funding to the University of Virginia for roles on Scientific Advisory Boards for Immatics and CureVac. Also, C.L.S. has received licensing fee payments through the UVA Licensing and Ventures Group (UVA LVG) for patents for peptides used in these cancer vaccines. Patents relevant to peptides in this vaccine include United States Patent # 6,660,276; 6,558,671; and 7,019,112; however, the terms of those patents have expired. He holds other patents for peptides in melanoma vaccines (US Patent number US 9,345,755 B2), plus one submitted, all managed by UVA LVG. There are no disclosures or competing interests for the other authors.

## ETHICS STATEMENT

The Mel39 trial was performed with approval by the University of Virginia Institutional Review Board (HIC#8878). All participants gave informed consent prior to participating in this trial. This trial is registered at Clinicaltrials.gov, identifier: NCT00938223.

## Supporting information


Data S1.

## Data Availability

De‐identified data from this clinical trial are available upon reasonable request from the corresponding author.
