# Supplementary material for: Twenty‐year survival outcomes after multipeptide vaccination for resected high‐risk melanoma: A post‐hoc analysis of a randomized clinical trial
Source: Int J Cancer. 2025 Jun 19;157(9):1912–23. doi: 10.1002/ijc.70006 (PMC12407048; doi:10.1002/ijc.70006)
Supplement: Supplementary file 1 — Data S1. [file IJC-157-1912-s001.pdf]

Supplementary Material

Title: Twenty-year survival outcomes after multipeptide vaccination for resected high-risk melanoma: a post-hoc analysis of a randomized clinical trial

Authors: Emily K. Ninmer, Hong Zhu, Kimberly A. Chianese-Bullock, Craig L. Slingluff, Jr.

Table of Contents

Supplementary Tables

Table S1. Summary of disease re-staging by AJCC 8th edition criteria
Table S2. Participant characteristics by age group
Table S3. Participant characteristics by sex
Table S4. Participant characteristics for on-study recurrences by vaccine arm
Table S5. Overall survival rates by 5-year intervals

Supplementary Figures

Figure S1. Clinical outcomes for the Mel39 study population
Figure S2. Survival outcomes by disease stage

Table S1. Summary of disease re-staging by AJCC 8th edition criteria

AJCC Stage, n (%)	Arm A (n=26)		Arm B (n=25)	
	6th Edition	8th Edition	6th Edition	8th Edition
IIA[a]	0 (0)	0 (0)	0 (0)	1 (4)
IIB	2 (8)	2 (8)	4 (16)	2 (8)
IIC	2 (8)	2 (8)	0 (0)	1 (4)
III NOS[b]	0 (0)	3 (12)	0 (0)	1 (4)
IIIA	9 (35)	3 (12)	8 (32)	3 (12)
IIIB	9 (35)	6 (23)	6 (24)	7 (28)
IIIC	1 (4)	6 (23)	3 (12)	5 (20)
IIID	-	1 (4)	-	0 (0)
IV[c]	3 (12)	3 (12)	4 (16)	5 (20)

AJCC, American Joint Committee on Cancer (8th edition); *NOS*, not otherwise specified

[a] One participant had stage IIA disease on re-staging by AJCC 8th edition criteria, but had stage IIB disease by AJCC 6th edition criteria, and thus was eligible for the trial.

[b] Participants with nodal disease and Tx tumor stage for which the stage category may be IIIB or IIIC depending on tumor thickness by 8th edition criteria.

[c] The additional participant classified as stage IV disease on arm B is due to incorrect staging in the original report. On review of source documents for re-staging, this participant was incorrectly classified as stage IIIB (by 6th edition criteria) in the original report, but should have been stage IV for distant skin metastases that were resected prior to enrollment.

Table S2. Participant characteristics by age group

	<65 years (n=37)	65+ years (n=14)	Total (n=51)	p value[a]
**Male**	23 (62%)	8 (57%)	31 (61%)	0.75
**Class I MHC allele[b], n (%)**				
**HLA-A1**	12 (32%)	4 (29%)	16 (31%)	1.00
**HLA-A2**	18 (49%)	10 (71%)	28 (55%)	0.21
**HLA-A3[c]**	18 (49%)	8 (57%)	26 (51%)	0.59
**Multiple HLA alleles[d], n (%)**	11 (30%)	8 (57%)	19 (37%)	0.07
**Disease status, n (%)**				0.98
**Initial diagnosis**	21 (57%)	8 (57%)	29 (57%)	
**Recurrent diagnosis**	16 (43%)	6 (43%)	22 (43%)	
**AJCC stage, n (%)**				0.72
**II**	6 (16%)	2 (14%)	8 (16%)	
**III**	24 (65%)	11 (79%)	35 (69%)	
**IV**	7 (19%)	1 (7%)	8 (16%)	
**ECOG PS score 0, n (%)**	31 (83%)	9 (64%)	40 (78%)	0.15

MHC, major histocompatibility complex; *HLA*, human leukocyte antigen; *AJCC*, American Joint Committee on Cancer (8th edition); *ECOG PS*, Eastern Cooperative Oncology Group Performance Status

[a] Chi-square test or Fisher's exact test (counts of n ≤ 5) for categorical variables; Mann-Whitney test for age; significant p < 0.05

[b] Counts represent the number of restricted alleles. Patients may express two different restricted alleles, resulting in counts greater than the sample size (> 100%).

[c] HLA-A3 superfamily, including HLA-A3, HLA-A11, HLA-A31.

[d] Counts represent the number of participants who express two different restricted alleles.

Table S3. Participant characteristics by sex

	Female (n=20)	Male (n=31)	Total (n=51)	p value[a]
**Median age, years (range)**	48.0 (28.2-82.1)	56.2 (36.0-73.5)	53.1 (28.2-82.1)	0.44
**Class I MHC allele[b], n (%)**				
**HLA-A1**	9 (45%)	7 (23%)	16 (31%)	0.10
**HLA-A2**	9 (45%)	19 (61%)	28 (55%)	0.26
**HLA-A3[c]**	8 (40%)	18 (58%)	26 (51%)	0.21
**Multiple HLA alleles[d], n (%)**	6 (30%)	13 (42%)	19 (37%)	0.39
**Disease status, n (%)**				0.35
**Initial diagnosis**	13 (65%)	16 (52%)	29 (57%)	
**Recurrent diagnosis**	7 (35%)	15 (48%)	22 (43%)	
**AJCC stage, n (%)**				0.29
**II**	3 (15%)	5 (16%)	8 (16%)	
**III**	16 (80%)	19 (61%)	35 (69%)	
**IV**	1 (5%)	7 (23%)	8 (16%)	
**ECOG PS score 0, n (%)**	15 (75%)	25 (81%)	40 (78%)	0.73

MHC, major histocompatibility complex; *HLA*, human leukocyte antigen; *AJCC*, American Joint Committee on Cancer (8th edition); *ECOG PS*, Eastern Cooperative Oncology Group Performance Status

[a] Chi-square test or Fisher's exact test (counts of n ≤ 5) for categorical variables; Mann-Whitney test for age; significant $p < 0.05$

[b] Counts represent the number of restricted alleles. Patients may express two different restricted alleles, resulting in counts greater than the sample size (> 100%).

[c] HLA-A3 superfamily, including HLA-A3, HLA-A11, HLA-A31.

[d] Counts represent the number of participants who express two different restricted alleles.

Table S4. Participant characteristics for on-study recurrences by vaccine arm

	Arm A (n=17)	Arm B (n=16)
**Median age, years (range)**	51.6 (28.2-75.0)	59.1 (40.2-82.1)
**Male, n (%)**	10 (59%)	14 (88%)
**Recurrent diagnosis (prior to enrollment), n (%)**	6 (35%)	10 (63%)
**AJCC stage of disease prior to enrollment, n (%)**		
**II**	4 (24%)	2 (13%)
**III**	11 (65%)	10 (63%)
**IV**	2 (12%)	4 (25%)
**CLND prior to enrollment, n (%)**	12 (71%)	9 (56%)
**PBMC immune response, n (%)**	10 (59%)	13 (81%)
**Death after enrollment, n (%)**	13 (76%)	10 (63%)
**Median time to first recurrence after enrollment, years (range)**	1.11 (0.23-8.56)	1.02 (0.02-15.27)
**Site of first on-study recurrence, n (%)**		
**Skin**	8 (47%)	6 (38%)
**Lymph nodes**[a]	3 (18%)	5 (31%)
**Lung**	3 (18%)	3 (19%)
**Brain**	2 (12%)	2 (13%)
**Unknown**[b]	1 (6%)	0 (0%)
**Therapies for first on-study recurrence**[c]**, n (%)**		
**Surgical resection**	12 (71%)	11 (69%)
**Systemic immunotherapy**[d]	2 (12%)	2 (13%)
**Cytotoxic chemotherapy**	3 (18%)	1 (6%)
**Radiation therapy**[e]	2 (12%)	1 (6%)
**Unknown**[b]	1 (6%)	0 (0%)
**Additional resectable on-study recurrence**[f]**, n (%)**	1 (6%)	4 (25%)
**Progressive metastatic disease, n (%)**	10 (59%)	7 (44%)

AJCC, American Joint Committee on Cancer (8th edition); *CLND*, completion lymphadenectomy; *PBMC*, peripheral blood mononuclear cell

[a] Includes regional and distant nodal metastases

[b] One participant had unknown site of recurrence and subsequent therapies, but was reported to have died of metastatic disease

[c] Excludes any subsequent therapies given for progressive metastatic disease

[d] Includes systemic interferon therapy and interleukin-2 therapy

[e] Includes gamma knife radiosurgery or whole brain radiation therapy; one participant with brain metastasis underwent surgical resection of the brain lesion

[f] Excludes participants found to have progressive metastatic disease

Table S5. Overall survival rates by 5-year intervals

	Overall Survival Rate (95% Confidence Interval)		
**Time Interval**	**Arm A (n=26)**	**Arm B (n=25)**	**Total (n=51)**
**5-year**	69% (51-87%)	76% (59-93%)	73% (60-85%)
**10-year**	62% (42-81%)	68% (49-87%)	65% (51-78%)
**15-year**	57% (37-77%)	64% (45-83%)	60% (47-74%)
**20-year**	38% (18-59%)	59% (40-79%)	49% (35-63%)

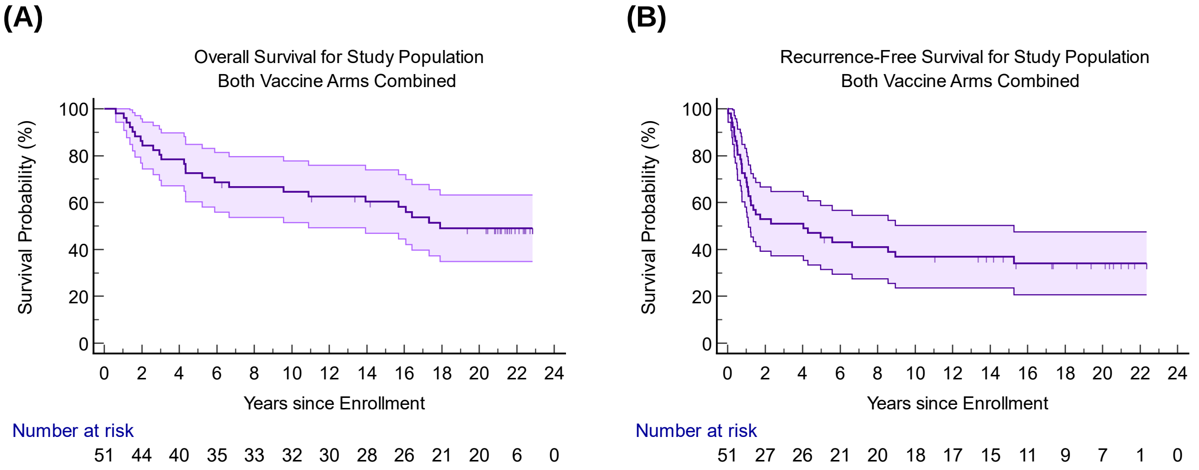

Figure S1. Clinical outcomes for the Mel39 study population. Kaplan-Meier curves for **(A)** overall survival (OS) and **(B)** recurrence-free survival (RFS) with 95% confidence intervals for the entire study population.

(A) **(B)**

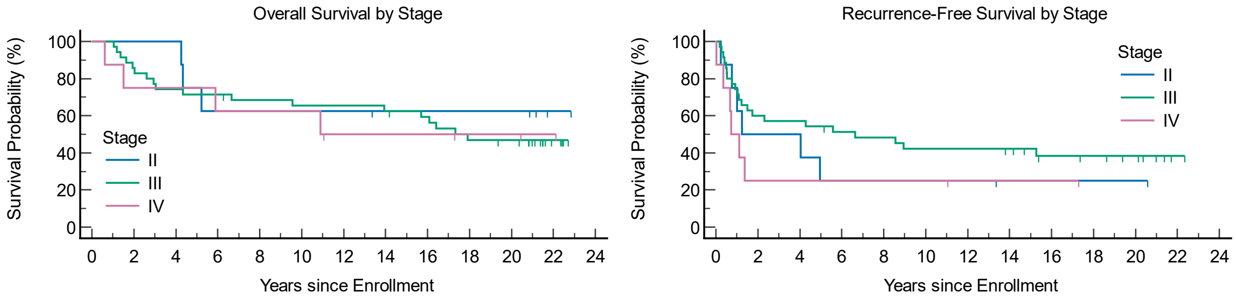

(A) Number at risk

Group	0	2	4	6	8	10	12	14	16	18	20	22	24
Group: II	8	8	8	5	5	5	5	4	4	4	4	1	0
Group: III	35	30	26	25	23	22	22	21	19	15	14	4	0
Group: IV	8	6	6	5	5	5	3	3	3	2	2	1	0

(B) Number at risk

Group	0	2	4	6	8	10	12	14	16	18	20	22	24
Group: II	8	4	4	2	2	2	2	1	1	1	1	0	0
Group: III	35	21	20	17	16	14	14	13	9	8	6	1	0
Group: IV	8	2	2	2	2	2	1	1	1	0	0	0	0

Figure S2. Survival outcomes by disease stage. Kaplan-Meier curves for **(A)** overall survival (OS) by American Joint Committee on Cancer (AJCC, 8th edition) stage (stage IV vs II: HR 1.62 95% CI: 0.39-6.75; stage IV vs III: HR 1.12 95% CI: 0.35-3.58; stage III vs II: HR 1.44 95% CI: 0.49-4.19; overall p=0.80), and **(B)** recurrence-free survival (RFS) by AJCC, 8th edition stage (stage IV vs II: HR 1.29 95% CI: 0.33-5.01; stage IV vs III: HR 1.89 95% CI: 0.63-5.64; stage III vs II: HR 0.68 95% CI: 0.26-1.82; overall p=0.32).
